# Using an Extension Catheter in Thrombus Aspiration for Coronary Subacute Myocardial Infarction

**DOI:** 10.1016/j.jaccas.2025.104135

**Published:** 2025-07-23

**Authors:** Yi-feng Zhou, Nan Feng, Zhao-Lin Zhang, Han-qing Zhao, Fei Lin, Zhi-gang Chen

**Affiliations:** a The First Affiliated Hospital of Xinxiang Medical College, Xinxiang, Henan, China; b Nanchang University Queen Mary School, Nanchang, China

**Keywords:** acute coronary syndrome, myocardial infarction, percutaneous coronary intervention

## Abstract

We report a case of subacute myocardial infarction thrombus aspiration using extension catheters. The presence of refractory thrombus was observed in all patients during coronary angiography, and favorable outcomes were achieved through the use of an extended catheter for thrombus extraction. In cases involving subacute or refractory thrombus, the efficacy of traditional thrombus aspiration catheters may be limited, necessitating the use of an extension catheter for effective thrombus removal.

## Graphical Abstract

**Figure:**
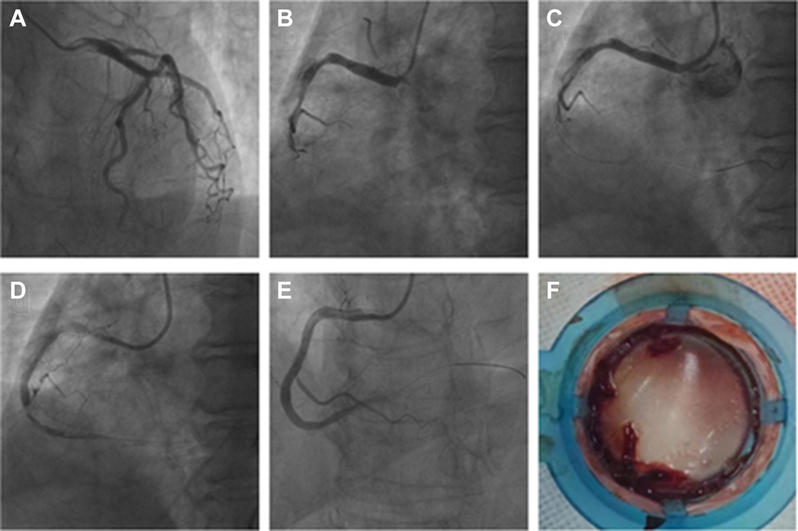

**Take-Home Messages**

• For subacute thrombi, conventional thrombus aspiration techniques tend to be less effective.
• The use of an extended catheter for subacute thrombus aspiration can achieve superior outcomes compared with conventional methods.

A 66-year-old man with risk factors for coronary heart disease, including hypertension, atrial fibrillation, smoking, and alcohol consumption, presented with episodic angina for 6 days that was related to activity. The initial electrocardiogram demonstrated atrial fibrillation and an inferior wall right ventricular ST-segment elevation myocardial infarction, leading to the diagnosis of SMI.

Coronary angiography revealed extensive collateral circulation from the left coronary artery to the right coronary artery (Figure 1A). The right coronary artery exhibited irregularities, with a discernible thrombus in the midsegment and complete distal occlusion (TIMI flow grade 0) (Figures 1B and 1C). Conventional thrombus aspiration using a standard catheter was ineffective (Figure 1D). Subsequently, an extension catheter with larger lumens was used (Table 1), successfully extracting a substantial amount of red thrombus (Figure 1F). Following this, balloon angioplasty and implantation of a rapamycin-eluting stent were performed at the distal lesions. Repeat angiography demonstrated no residual stenosis and restored TIMI flow grade 3 (Figure 1E).

**Figure 1:**
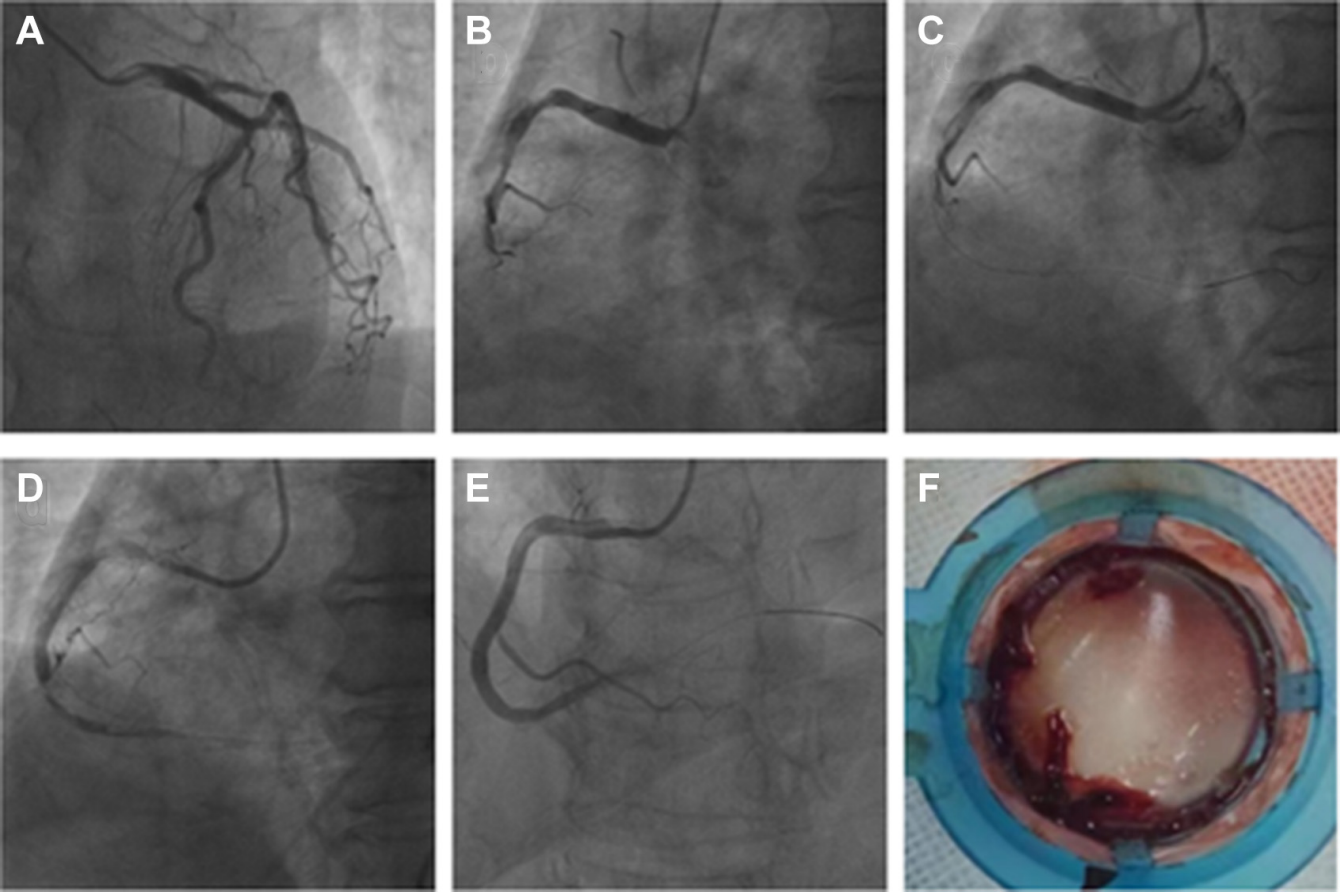
Coronary Angiography (A) Initial left coronary artery. (B,C) Initial right coronary artery. (D) After normal thrombus suction. (E) After extension catheter suction. (F) Massive subacute intracoronary thrombus and plaque obtained by extension catheter aspiration.

**Table 1:** Details of Catheters

	Internal Diameter, in	External Diameter, in
Guide catheter (6-F)	0.071	
Thrombus aspiration catheter (6-F)	0.044	0.067
Extension catheter (6-F)	0.056	0.067

All catheters were provided by Medtronic.

Currently, the treatment of patients with SMI with significant thrombus burden poses substantial clinical challenges. At this stage, the thrombus is partially organized but not fully consolidated, rendering it more malleable compared with an acute thrombus.^1^^,^^2^ Direct interventional therapy carries the risk of dislodging the thrombus, leading to distal vessel occlusion, while thrombolytic agents are often ineffective. Routine catheter thrombectomy frequently results in residual refractory thrombi. The extension catheter, commonly used to address high resistance during instrument delivery, features a large aperture and high negative pressure, which enhances thrombus aspiration in SMI cases.^3^ Clinical experience indicates that for patients with SMI with high thrombus burden, initial suction using a conventional catheter can be followed by extension catheter aspiration if necessary. This approach has consistently improved outcomes, reducing thrombus burden and enhancing distal blood flow. It is important to emphasize that during the process of thrombus aspiration using an extension catheter, continuous aspiration should be performed until smooth flow is achieved. Additionally, following the removal of the extension catheter, the guide catheter should be reused and subjected to aspiration once more to prevent the potential retention of residual clots. This can effectively prevent the occurrence of thrombus shedding into the systemic circulation, thereby avoiding associated adverse events.

For subacute or refractory thrombi, for which conventional catheters are inadequate, the extension catheter effectively removes thrombi, thereby avoiding potential complications associated with stent therapy due to coronary artery flow disorders.

## Funding Support and Author Disclosures

The authors have reported that they have no relationships relevant to the contents of this paper to disclose.
